# An Updated Review of Interventions that Include Promotion of Physical Activity for Adult Men

**DOI:** 10.1007/s40279-014-0286-3

**Published:** 2014-11-28

**Authors:** Joan L. Bottorff, Cherisse L. Seaton, Steve T. Johnson, Cristina M. Caperchione, John L. Oliffe, Kimberly More, Haleema Jaffer-Hirji, Sherri M. Tillotson

**Affiliations:** 1 Institute for Healthy Living and Chronic Disease Prevention, and School of Nursing, University of British Columbia, ART223, 3333 University Way, Kelowna, BC V1V 1V7 Canada; 2 Faculty of Health Sciences, Australian Catholic University, Victoria, Australia; 3 Faculty of Health Disciplines, Athabasca University, Athabasca, AB Canada; 4 School of Health and Exercise Science, University of British Columbia, Vancouver, BC Canada; 5 School of Nursing, University of British Columbia, Vancouver, BC Canada; 6 Northern Health, Prince George, BC Canada

## Abstract

The marked disparity in life expectancy between men and women suggests men are a vulnerable group requiring targeted health promotion programs. As such, there is an increasing need for health promotion strategies that effectively engage men with their health and/or illness management. Programs that promote physical activity could significantly improve the health of men. Although George et al. (Sports Med 42(3):281, 30) reviewed physical activity programs involving adult males published between 1990 and 2010, developments in men’s health have prompted the emergence of new sex- and gender-specific approaches targeting men. The purpose of this review was to: (1) extend and update the review undertaken by George et al. (Sports Med 42(3):281, 30) concerning the effectiveness of physical activity programs in males, and (2) evaluate the integration of gender-specific influences in the content, design, and delivery of men’s health promotion programs. A search of MEDLINE, CINAHL, ScienceDirect, Web of Science, PsycINFO, the Cochrane Library, and the SPORTDiscus databases for articles published between January 2010 and August 2014 was conducted. In total, 35 studies, involving evaluations of 31 programs, were identified. Findings revealed that a variety of techniques and modes of delivery could effectively promote physical activity among men. Though the majority of programs were offered exclusively to men, 12 programs explicitly integrated gender-related influences in male-specific programs in ways that recognized men’s interests and preferences. Innovations in male-only programs that focus on masculine ideals and gender influences to engage men in increasing their physical activity hold potential for informing strategies to promote other areas of men’s health.

## Key Points

**Table:** 

“Gender-sensitized” physical activity programs are a key development in men’s health promotion and demonstrate potential for engaging hard-to-reach men.
Four programs that engaged men through organized sports resulted in increased physical activity.
Programs with a diverse set of components, including online and mobile platforms, may impact the physical activity of men if the approach is simple, clear, and tailored to men’s interests and preferences.

## Introduction

Epidemiological evidence clearly supports the role that physical activity plays in health promotion and disease prevention. Regular physical activity (which includes exercise) reduces the risk not only of premature mortality, but also coronary heart disease, hypertension, some cancers, type 2 diabetes, high body mass and mental illness [1–4]. These benefits extend beyond primary prevention, and when combined with other healthy lifestyle behaviors (e.g., healthy eating), can add up to 14 years to life expectancy for people living with chronic disease [5]. This is particularly important for men, who, compared to their female counterparts, are more likely to have a shorter life expectancy and experience higher mortality rates associated with chronic disease [6–8]. Despite the health benefits associated with physical activity, research has highlighted that an alarming proportion of men do not engage in the recommended levels of physical activity (150 min or more of moderate intensity physical activity per week) for optimal health benefits [9, 10]. Globally, approximately 28 % of men are insufficiently active, with the highest prevalence in the Americas (40 %) and Eastern Mediterranean regions (36 %) [11]. Recent interest in men’s health, particularly in relation to addressing and preventing obesity, has led to a number of reviews outlining effective approaches towards risk reduction through active living and healthy eating [12–14]. Within these reviews are well-defined considerations around effective modes of delivery and the use of behavioral theory to support adoption of these behaviors.

Health professionals routinely point to males as a ‘hard-to-reach’ population wherein unique challenges reside for implementing illness prevention and health promotion initiatives such as physical activity [15–17]. Specifically, males are less willing than their female counterparts to have an annual health check or seek advice from a health professional, less willing to attend health education sessions, and are less interested in information concerning illness and disease prevention compared to women [15, 18–22]. In terms of research, a clear gap remains in which little male-specific research concerning health promotion is available. Intervention research addressing physical activity has predominately included female samples [23–26], making it difficult to translate these outcomes to males. These inequities are even more pronounced among sub-groups of men (e.g., low socio-economic status, Aboriginal, immigrant men) and raise questions concerning sex and gender influences in developing targeted approaches to men’s health promotion programs and interventions [27].

Although researchers have examined the effectiveness of physical activity interventions [28], few have focused on program effects by sex or considered the influence of gender-related factors [29]. One recent review by George et al. [30] provided a critical evaluation of studies examining the effectiveness of physical activity programs in adult males. They found that only a limited number of physical activity interventions targeted men specifically. Although promising modes of delivering programs to men were associated with improved male participation rates, retention, and increased overall success, there was little focus on examining physical activity interventions for men in relation to the influences of gender and the role of masculinities. Theories of masculinity and increasing attention to sex and gender influences on health and health behavior have prompted explorations and new understandings of men’s health in recent years [31, 32]. These developments have led to the emergence of new sex- and gender-specific health promotion approaches targeting men [15, 33].

The two-fold purpose of the current review was, therefore, to: (1) extend and update the work undertaken by George et al. [30] concerning the effectiveness of physical activity programs in males, and (2) evaluate the integration of sex- and gender-related influences in the content, design, and delivery of these men’s health promotion programs.

## Literature Search Methodology

A comprehensive review of physical activity programs involving men was undertaken between January 2010 and August 2014. The databases MEDLINE, CINAHL, ScienceDirect, Web of Science, PsycINFO, The Cochrane Library, and SPORTDiscus were searched using all combinations of the terms ‘male or men’ with ‘physical activity, exercise, or sport’ and ‘intervention, program, or trial’. No unpublished or grey literature was searched. Two research assistants (KM and HJH) completed the initial search and a project coordinator (CS) oversaw the process to ensure the search strategies and exclusion criteria were consistently followed.

Replicating George et al.’s [30] inclusion criteria, we used the following criteria to identify articles for this review. All study designs were included (e.g., randomized controlled trial [RCT], pre-post, quasi-experimental, etc.) provided they met the following: (1) included only male participants or data that was disaggregated by sex; (2) all participants were aged 18 or older; and (3) at least one of the outcomes of the study was a change in physical activity (and exercise) or a measure of biomarkers of disease related to a change in physical activity (e.g., body mass index [BMI], blood pressure, waist circumference). Articles that included both physical activity and other health behaviors (e.g., diet) were included, provided they met the aforementioned criteria. Only articles published in English were considered. Additionally, articles with participants who were solely 65 years and older were excluded. If more than one article was published based on the same sample of participants, the most relevant article was retained for this review (no secondary publications).

**Fig. 1:**
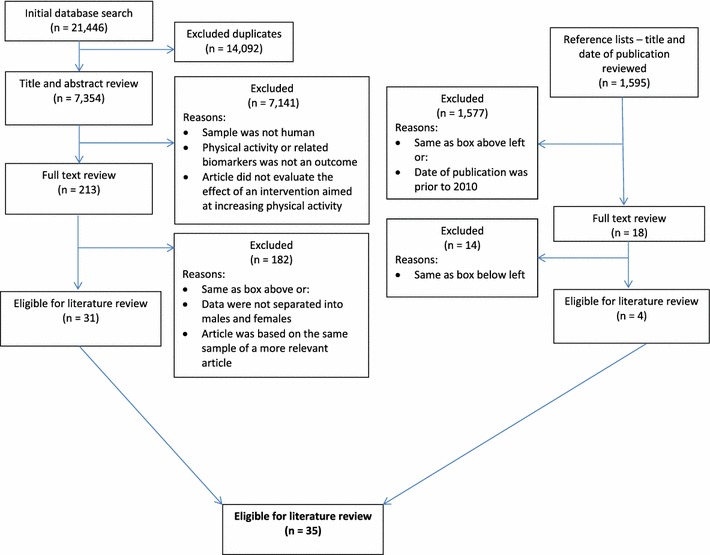
Pathway of articles identified and excluded

Our search yielded a total of 21,446 articles and resulted in 7,354 articles after duplicates were removed using RefWorks. A title and abstract review was conducted (KM and HJH) to exclude articles that did not meet the eligibility criteria. In total, 213 articles were identified for further assessment, and the full texts of these articles were reviewed (KM and HJH). After excluding articles that did not meet the inclusion criteria (e.g., did not report results separated by sex), as well as those that were based on the same sample (*n* = 4), 31 articles were identified for inclusion. The reference lists of the articles that met the inclusion criteria were examined, and of the additional 1,595 articles evaluated, four met the inclusion criteria. A flow diagram summarizing article inclusion/exclusion is provided in Fig. 1. Altogether, the search resulted in 35 articles identified for inclusion: 13 articles reported on evaluations of programs focusing on physical activity only (Table 1), and 22 reported on evaluations of programs that included both physical activity and other health behaviors (e.g., healthy eating) (Table 2). In the presented results, programs that were evaluated in more than one study were counted once in summaries of program details.

**Table 1:** Summary of the studies focused on physical activity alone in adult men from January 2010 to August 2014

Study	Study design	Participants	Theoretical basis	Gender focus?	Mode of delivery	Outcomes	Program	Program duration	Changes in PA and other secondary outcomes
Andersen et al. [34]	RCT (with 6 months follow-up post-program end)	150 Pakistani immigrant men 25–60 years Norway	Social cognitive theory	Male-only sample	Community-based Group-based Face-to-face	PA measured by an accelerometer (CPM), minutes of sedentary behavior, and minutes of light, moderate, vigorous, and very vigorous PA	The Physical Activity and Minority Health study included two groups:	5 months	Changes in PA:
									Yes. Total PA was higher in intervention group compared to the control group at 6 month follow-up
							PA program group (consisted of group exercise sessions twice per week, two group lectures, one individual counselling session, written materials, and a phone call)		
							Control group (received their baseline results only. After the intervention the group was offered the chance to participate in organized exercise once/week for 4 months, one group lecture, and written materials)		
									Other outcomes:
									Changes in social cognitive constructs (self-efficacy, social support and outcome expectancies) did not mediate changes in PA
Baruth et al. [68]	RCT	874 Inactive adults (479 men) recruited from primary care facilities (e.g. physician’s office) 35–74 years United States	Social cognitive theory and transtheoretical model	Co-ed	Individual-based Print-based Telephone-based Face-to-face	BMI, lipids and lipoproteins (TC, HDL-C, LDL-C and TG), SBP, DBP, and cardiorespiratory fitness	The Activity Counselling Trial (ACT) intervention included three treatment groups: Advice (standard care: received 2–4 min of counselling on the recommended amount of PA and were given written material about recommended PA guidelines. The advice group were also allowed to call a health educator about any questions regarding PA) Assistance group (given all the same materials and resources as the advice group, as well as a 30–40 min counselling session with a health educator, monthly interactive mail newsletters, a pedometer and a calendar) Counselling group (received everything that the advice and assistance groups received. Additionally the counselling group also received biweekly telephone counselling for the first 6 months, monthly telephone counselling for the remainder of the first year and telephone counselling for the final year. The frequency was determined by the participant and the counsellor)	2 yr	Changes in PA: Could not determine Other outcomes: There were no significant differences between the three treatment groups. In men (across all groups) there was a significant decrease in DBP, TC, LDL-C cholesterol, the total cholesterol to HDL-C cholesterol ratio and TG from baseline at 24 months. For men SBP significantly decreased if their weight also significantly decreased
Dalleck et al. [62]	Pre-post	332 Adults (142 men) 28–88 years United States	No	Co-ed	Community-based Individual-based Face-to-face	Absolute and relative energy expenditure, WC, body mass, SBP, DBP, TC, HDL-C, LDL-C, TG, blood glucose, cardiorespiratory fitness, and 10 year risk score	Exercise program (each participant worked with an exercise trainer 3 days/week for 14 weeks and aimed to reach an individualized energy expenditure of 14–23 kcal kg^−1^ week^−1^. The exercise trainer coached and provided support for the participant)	14 weeks	Changes in PA: Could not determine. Participants engaged in supervised exercise and outcomes related to cardiovascular risk were measured, but PA was not measured as an outcome Other outcomes: All outcomes (except total cholesterol) and 10-year cardiovascular risk score improved significantly following the program
Foulds et al. [35]	Pre-post	273 Aboriginal adults (58 men) 18–75 years Canada	No	Co-ed	Community-based Group-based Face-to-face	PA, weight, BMI, WC, SBP, DBP, TC, HDL-C, TC:HDL-C ratio, and blood glucose	Hearts in Training (participants selected one of three PA interventions: walking, walk/running, or running to train for the Vancouver Sun Run, and received weekly group training sessions in local Aboriginal community as well as individual training twice per week)	13 weeks	Changes in PA: Yes. PA scores improved with training in all groups Other outcomes: WC, SBP, and total cholesterol improved with training. Most consistent improvements were seen in the walking group, who scored worse than the walk/running and running groups pre-intervention. Participants appropriately self-selected PA programs suited to their ability
Gany et al. [56]	Pre-post pilot study	74 South Asia taxi drivers 30–79 years United States	No	Male-only sample	Individual-based Paper-based Telephone-based	Daily step counts, SBP, DBP, LDL-C, HDL-C, blood glucose, and BMI	Supporting South Asian Taxi Drivers to Exercise through Pedometers (SSTEP; included pedometers, written materials and a logbook for tracking steps along with telephone follow-up)	12 weeks	Changes in PA: Yes. Participants in SSTEP program increased their daily steps from baseline Other outcomes: No significant changes in any of the secondary measures. Participants with higher baseline blood glucose had greater increases in step counts
Gram et al. [69]	RCT	61 Caucasian men who were sedentary and moderately overweight with no first degree relatives with type 2 diabetes 20–40 years Denmark	Theory of planned behavior	Male-only sample	Group-based Face-to-face	Body weight, BMI, *V*O_2max_, mL O_2_, fat free mass, fat mass, and attitude towards PA	In Project FINE (Four-IN-onE; participants were assigned to be a part of the control group, the moderate intensity exercise group (300 kcal a day/30 min), or the high intensity exercise group (600 kcal a day/60 min). Exercise was supervised using a heart rate monitor and through meetings with the project’s research staff. A subset of participants from each group participated in semi-structured interviews)	12 weeks	Changes in PA: Yes. The intervention included supervised PA of both moderate and high intensity. Adherence to PA was higher in moderate intensity group Other outcomes: Both the moderate and high intensity group, but not the control group, lost body weight, lowered their BMI, and improved their *V*O_2max_, and mL O_2_ ratio. Body mass composed of fat decreased in both the exercise groups but not the control group. Participants that were part of the high intensity exercise group perceived 60 min of PA a day to be too tiring and time consuming. Subjects that were part of the moderate intensity exercise group reported an increased awareness about lifestyle habits and their health conditions. Further, they had a positive attitude towards exercise and a perceived increase in energy
Griffith et al. [70]	Pre-post pilot study	41 African American men who reported having a physician’s approval to participate in an intervention aimed at increasing PA 35–70 years United States	Social cognitive theory and self determination theory	Male-only sample and male-centred approach The program involved small group, male-focused PA, with age-appropriate role modelling and male peer support	Face-to-face Community-based	WC, weight, blood pressure, TC, HDL-C. LDL-C, glucose levels, and TG	Pilot study of the *Men on the Move* program (groups of 5–10 men met with a personal trainer once a week for 10 weeks. During the group sessions they participated in PA (e.g., flexibility, strength and cardiovascular exercises). The intervention aimed to teach men how they can engage in PA anywhere at a low cost. Additionally, participants were provided with a list of PA classes that were being held in their area, a list of websites that were thought to be helpful in aiding men increase PA, and a contact list of their group members (to encourage getting together outside of scheduled group sessions)	10 weeks	Changes in PA: Yes. Self-report measures showed significant improvements from baseline in participants’ overall PA level, intensity of PA, and motivation to engage in PA Other outcomes: Significant improvements from baseline in health status, and stress level. Further, all of the physiological variables improved, however these improvements were not statistically significant
Hooker et al. [38]	Pre-post	25 African American Men 45–66 years United States	Social cognitive theory	Male-only sample and male-centred approach The program included cultural and gender sensitive (tailored to masculine identity) elements based on formative research	Group-based Face-to-face	Self-reported PA, body weight, lower-body leg strength, flexibility, aerobic fitness, social support, self-efficacy, self-regulation (for PA), and participant satisfaction with the program	Intervention (consisted of 90 min group sessions twice/week. Session content included friendly competition and camaraderie, PA goals recorded weekly, and most PA was conducted outside of sessions)	8 weeks	Changes in PA: Yes. Significant positive changes for PA Other outcomes: Self-efficacy, social support, self-regulation, functional fitness and aerobic fitness significantly improved. Very high participant satisfaction with program. The research staff reported that friendly competition suited men well
Kim et al. [39]	Pre-post	376 Male employees with at least 1 metabolic risk factor 30–62 years Japan	No	Male-only sample	Individual-based Internet-based (email) Face-to-face Workplace-based	BMI, PA, WC, SBP, DBP, TG, fasting plasma glucose, and HDL-C	Workplace Lifestyle-based Physical Activity Intervention program (participants aimed to walk 3,000 steps/day on at least 5 days/week and 2,000 of the steps were to be obtained through brisk walking. Participants also received a 1.5 h lecture on the benefits of PA and pedometer use, and received monthly reports on adherence to program)	1 year	Changes in PA: Yes. Both total steps and brisk walking were significantly increased at 1 year follow-up Other outcomes: A reduction in metabolic syndrome risk factors was correlated with an increase in the brisk walking step count and brisk walking for at least 10 min was advantageous for improving metabolic syndrome. Significant improvements were observed in WC, SBP, HDL-C, and fasting plasma glucose The prevalence of metabolic syndrome was reduced from 39.9 to 20.9 %
Martin-Valero et al. [63]	RCT pilot study	75 Inactive adults with cardiovascular risk factors (31 men) 57–69 years Spain	No	Co-ed	Group-based Face-to-face	Weight, height, SBP, DBP, heart rate, rated perceived effort, forced vital capacity, forced expiratory volume in one second, and physical and mental health	The Physical Activity Promotion Program (PAPP) included two groups: Intervention group (60 min exercise session including a warm up, aerobic exercise, and a cool down twice/week for 12 weeks) Control group (received educational leaflet)	12 weeks	Changes in PA: Could not determine Did not measure PA as an outcome, but assigned intervention group to 60 min exercise sessions Other outcomes: Significant improvement in pulmonary outcomes (except forced expiratory volume in one second) and in the quality of life of men in the intervention group
Plotnikoff et al. [58]	RCT trial (with 6 months follow-up post-program end)	287 Adults with type 2 diabetes mellitus (154 men) Canada	Integrated stage approach (described as a combination of social-cognitive constructs, items from the theory of planned behavior, transtheoretical model, social cognitive theory, motivation theory, and the health belief model)	Co-ed	Individual-based Print-based Telephone-based	PA (self-reported and steps), glycosylated hemoglobin (A_1c_), insulin, glucose, TC, HDL-C, LDL-C, TG, and health related quality of life (physical and mental health scale)	The Alberta Diabetes and Physical Activity Trial (ADAPT) included three trial groups: Print material group (PA guidelines by the Canadian diabetes association, stage based print materials, pedometer, logbook, and calendar) Telephone counselling group (received the same materials as the print materials group as well as follow-up telephone calls from trained counsellors for additional support) Control group (received PA guidelines and pedometer, but was instructed not to use pedometer between the assessment periods)	1 year	Changes in PA: No. PA did not change in intervention group versus control group Other outcomes: Clinical variables did not change for men in the intervention groups versus the control group The health related quality of life physical scale scores increased significantly in the intervention groups compared to the control group. Combined print materials, pedometer, and telephone counselling was ineffective for increasing men’s PA
Sheeran et al. [40]	RCT (with 6 months follow-up post-program end)	467 Male members of a fishing club Mean age: 53.88 years England	Fantasy realization theory and mental contrasting along with variables based on the theory of planned behavior	Male-only sample	Individual-based Telephone-based Face-to face	Self-reported level of PA and variables based on the theory of planned behavior (i.e., perceived behavioral control, subjective norms, and attitudes)	Gone Exercising (consisted of a double-blind randomized controlled trial in which the participants were either assigned to the control group or the mental contrasting intervention group. A baseline questionnaire was administered to both groups and consisted of demographics, reporting PA, and measured planned behavior relating to PA. Participants that were part of the intervention group received a mental contrasting induction to prompt them to mentally work through the barriers to engaging in PA. Participants were telephoned 1 month after baseline measurement and 7 months after baseline measurement to obtain measure of level of PA)	1 month	Changes in PA: Yes. Mental contrasting intervention enhanced rates of self-reported PA Other outcomes: Mental contrasting enabled participants to translate their attitudes about the importance of PA into action (i.e., engaging in PA)
Young et al. [64]	RCT	378 (197 men) individuals with abnormal lipid profiles 30–64 years for men United States	No	Co-ed	Group-based Face-to-face	Plasma high-sensitivity, HDL-C, LDL-C, TG, fasting blood glucose, and WC	The Diet and Exercise for Elevated cardiovascular disease Risk (DEER; individuals were randomly assigned to be part of the control group, PA only group, low-fat diet only group, or low-fat diet and PA group. In the low-fat diet groups, participants were provided with one individualized counselling session that was conducted by a registered dietician, followed by 8 group sessions. After the 12 week adoption phase participants received monthly follow ups for the following 6–8 months. Participants in the PA groups met with an exercise coach on week one and were offered 3 group classes a week during the 12 week activation phase. Participants were also offered group exercise options for 7–8 months during the maintenance phase. Participants were supposed to engage in an equivalent to 10 miles a week of fast walking or jogging)	1 year	Changes in PA: Could not determine Did not measure PA directly Other outcomes: For the male participants a change in blood pressure and TG was associated with a change in plasma high-sensitivity. For men that were part of the low-fat diet plus PA group a change in plasma high sensitivity was associated with a change in the percentage of their body mass that is composed of fat as well as a change in their fasting insulin

*BMI* body mass index, *Co-ed* coeducational, meaning open to both sexes, *CPM* average counts per minute per day, *DBP* diastolic blood pressure, *HDL-C* high-density lipoprotein cholesterol, *LDL-C* low-density lipoprotein cholesterol, *mL O*_*2*_ millilitres of oxygen in the blood, *PA* physical activity, *Pre-post* pre-intervention assessment and post-intervention assessments were conducted, *RCT* Randomized controlled trial, *SBP* systolic blood pressure, *TC* total cholesterol, *TG* triglycerides, *VO*_*2max*_ Maximum volume of oxygen, *WC* waist circumference

**Table 2:** Summary of the studies focused on physical activity along with other health behaviors in adult men from January 2010 to August 2014

Study	Study design	Participants	Theoretical basis	Gender focus?	Mode of delivery	Outcomes	Program	Program duration	Main findings
Aadahl et al. [41]	RCT	Study population: 10,108 (4,870 men) 30–60 years Denmark	Social cognitive theory, health belief model, and transtheoretical model	Co-ed	Individual-based Face-to-face	PA	The Inter99 study included 3 groups: High intensity intervention (Individual lifestyle counselling based on personal risk estimate and motivation for change. The initial 15–45 min lifestyle consultation focused on PA, along with smoking, diet and alcohol. Participants were encouraged to exercise for a minimum of 30 min/day. Individuals with high risk for ischemic heart disease were offered group counselling on diet, PA, and smoking cessation/reduction) Low intensity intervention (Individual lifestyle counselling and individuals with high risk for ischemic heart disease referred to GP) Control group (no intervention)	5 years	Changes in PA: Significant beneficial effect on PA over 5 years of intervention for men in high intensity intervention Other outcomes: Differences in PA across intervention group were not related to level of education
Borel et al. [42]	Pre-post design as well as comparison to a reference group of non-obese men	144 Viscerally obese men with insulin resistance and reference group of 47 non-obese men 30–65 years Canada	No	Male-only sample	Individual-based Face-to-face	Weight, hip circumference, WC, fat mass, fat free mass, excess visceral adipose tissue, subcutaneous abdominal adipose tissue, cardiorespiratory fitness, plasma glucose, plasma insulin, plasma lipoprotein, lipid profile, and adipokine inflammatory markers	SYNERGIE intervention group (Participants were given counselling with a nutritionist and a kinesiologist once every two weeks for the first 4 months of the intervention, followed by monthly counselling for the remainder of the year. The nutritional counselling aimed for the participants to have a 500 kcal daily energy deficit. The PA counselling aimed for the participants to engage in a minimum of 160 min of moderate intensity endurance exercise per week. The participants received a personalized training program and had free access to a fitness center) Reference group (baseline assessments were taken from a group of non-obese, healthy men to serve as a reference/target)	1 year	Changes in PA: Yes. PA significantly increased in the intervention group Other outcomes: After the 1-year intervention men in the intervention group had lost weight (average = 7 kg), and the visceral adipose tissue decreased by 18.4 % and subcutaneous abdominal tissue decreased by 21 % in this group Cardiorespiratory fitness significantly increased in the intervention group and daily caloric intake significantly decreased. The BMI was still higher in the intervention group compared to the reference group but their SBP, DBP and HR matched the levels of the reference group
Brady et al. [61]	Pre-post pilot study (with 15 months follow-up post-program end)	40 Adult men obtained from the Rangers and Celtic football team season ticket holder databases 40–60 years Scotland	No	Male-only sample and male-centered approach. The exercise program held at two football clubs was designed to harness men’s dedication as fans of football and was supervised by football coaches	Group-based Face-to-face	Body weight, SBP, DBP, TC, and blood glucose levels	Glasgow Celtic and Glasgow Rangers Football Clubs exercise program (was individually tailored to find the participants ideal heart rate level to reach during exercise. Heart monitors were used to observe heart rate and participants were asked to exercise for 20 min at their ideal heart rate 3–4 times a week. There were ten weekly 2 h group based sessions The first hour of each session was a health seminar taught by a physician. The second half of the group based sessions consisted of group exercise activities; these sessions were supervised by professional football coaches)	10 weeks	Changes in PA: Could not determine. Did not measure increases in PA directly Other outcomes: After the 10 week intervention there was a significant positive change in the participant’s cardiovascular health. The average weight lost per participant was 2.73 kg (4 % reduction in total body weight). There was a small decrease in SBP but DBP did not change. TC was reduced by 8 %. The 15 month follow up (*n* = 36) revealed that the weight loss that was originally achieved by the participants was maintained. Additionally, on average men had lost another 1.05 kg. SBP and DBP levels did not change at the follow up
Duncan et al. [55]	RCT	317 Men 35–54 years Australia	Social cognitive theory and self-regulation theory	Male-only sample and male-centered approach The program was tailored specifically to men based on research regarding men’s preferences and a review of other published interventions targeting men	Individual-based Internet-based Print-based	Self-reported minutes of PA, self-reported sessions of PA, dietary behaviours, health literacy, satisfaction with program, and use of information technology platform	The ManUp program included two groups: Information-technology-based intervention arm (participants had access to an online platform to record and view their progress, review educational information, and connect with online friends. There was also a mobile phone application available to participants with cellphones) Print-based intervention arm (participants received hard copy educational materials and a print booklet to track their progress)	9 months	Changes in PA: Yes. PA (minutes and sessions of PA) was increased in both the print-based and the information-technology-based intervention groups Other outcomes: Dietary behaviours were significantly improved in both groups, health literacy was improved, and over half of the participants in each group were satisfied with the PA challenge. Use of the information technology platform was not associated with PA or dietary behaviour
Eto et al. [43]	Pre-post	50 Men with BMI >25 kg/m^2^ 40–64 years Japan	No	Male-only sample	Group-based Face-to-face	Body weight, BMI, fat mass, fat-free mass, % fat mass, intra-abdominal fat area, subcutaneous fat area, SBP, DBP, TC, HDL-C, LDL-C, TG, Fasting plasma glucose, HbA1c, *V*O_2peak_, total energy intake, and PA	Free-living Physical Activity Promotion Weight Loss Program (consisted of 2 phases. First, the 3 month diet modification program included weekly (90 min) instructional sessions, individual counselling, lectures on low-calorie diets and eating behaviors. Participants kept food diary and received individualized feedback Second, the 3 month PA promotion phase included weekly (90 min) sessions consisting of lectures (30 ins) and walking (60 mins). Participants were instructed to continue PA over the week and target a total of 250 min/week of moderate-vigorous PA)	6 months	Changes in PA: Yes. PA increased following PA phase Other outcomes: Significant reduction in body weight, BMI and percentages of fat mass, intra-abdominal fat area and subcutaneous fat area, and improvements in HDL-C, and *V*O_2_peak following diet phase (3 month). Significant decrease in energy intake, fat-free mass, SBP, DBP, TG, fasting plasma glucose, and HbA1c following diet phase but no additional change following PA phase. Significant decrease in TC and LDL-C following diet phase but these returned to baseline following PA phase
Freak-Poli et al. [44]	Pre-post	762 Adults who were sedentary during their work hours (303 males) Mean age: 39.8 years Australia	No	Co-ed	Group-based Face-to-face Internet-based Workplace-based	Diabetes risk factors, cardiovascular disease risk factors, PA, TC, WC, SBP, DBP, and fruit and vegetable intake	The intervention was part of the Global Corporate Challenge (GCC; the Global cooperate challenges is a 125 day work place intervention that uses pedometers as a tool and encourages participants to reach 10,000 steps a day. Employees are broken up into teams of 7 people who enter their step counts online to “virtually walk around the world”. Weekly e-mails are sent out to all participants to motivate them to reach the prescribed step count. The Global Corporate Challenge website also includes additional health related information, forums, and a function to compare your teams progress with the progress of other teams)	4 months	Changes in PA: Yes. Male participants increased from baseline in meeting the recommended guidelines for PA Other outcomes: Male participants increased from baseline in meeting the recommended guidelines for fruit and vegetable intake. SBP and DBP was also significantly improved. Male participants decreased from baseline in TC and TG. Finally, male participants decreased their risk from baseline of being diagnosed with diabetes within the next 5 years
Gray et al. [45]	RCT pilot study (with 3 and 9 months follow-ups post-program end)	103 Men with a BMI of 27 kg/m^2^ or greater 35–65 years Scotland	No	Male-only sample and male-centered approach The program was tailored specifically to men by promoting peer support, using behavior change techniques, and encouraging competition and humor	Group-based Face-to-face	Weight, height, BMI, WC, resting BP, body fat, self-reported PA and diet, and alcohol intake	Pilot Football Fans In Training (FFIT; coaching staff from the Scottish Premier League delivered 12, 90 min sessions that included health education and PA components. Participants learned about healthy eating and PA as well as received tailored aerobic, strength, and flexibility training exercises. Participants aimed to walk for 45 min per day)	12 weeks	Changes in PA: Yes. Participants significantly increased their moderate-vigorous PA Other outcomes: Participants in the intervention group reduced their WC and BP, and 45.5 % of participants achieved a 5 % decrease in body weight at 12 weeks. These participants also improved their diet The improvements in weight loss, WC, PA, and diet were maintained at 6 and 12 months The FFIT program adequately recruited and retained members of the target population
Hunt et al. [46]	RCT (with 9 months follow-up post program end)	747 Men with a BMI of 28 kg/m^2^ or greater 35–65 years Scotland	No	Male-only sample and male-centered approach The program was gender sensitised in context (i.e., delivered to football fans in professional football clubs), drew on peer-support, and included content tailored specifically for men	Group-based Face-to-face	Weight, height, BMI, WC, resting BP, body fat, self-reported PA and diet, alcohol intake, and psychological outcomes	Football Fans In Training (FFIT; coaching staff delivered weekly 90 min sessions for 12 weeks to groups of up to 30 men in 13 different Scottish football clubs. After the initial 12 weeks, a 9 month maintenance stage included email prompts to participants and one group reunion) Control group (1 year waitlist)	12 weeks	Changes in PA: Yes. Significantly higher self-reported PA in intervention group compared to control group Other outcomes: Participants in the intervention group reduced their weight, BMI, WC, BP, and body fat significantly more than those in the control group. They also improved their diet and psychological outcomes
Jakicic et al. [47]	RCT	3,942 Overweight or obese individuals with type 2 diabetes (1,603 men) United States	No	Co-ed	Group-based Face-to-face	Height, weight, BMI, WC, cardiorespiratory fitness, leisure time PA, and glycemic control	Intensive Lifestyle Intervention (ILI; month 1–6: group and individual sessions. Month 7–12: 2 group sessions/month, 1 individual session/month, 1 motivational campaign. Month 13–48: monthly in person meeting with counsellor plus contact through email or telephone and 2 refresher campaigns For the dietary component, participants were instructed to aim for an energy intake of 1,200–1,800 kcal/day with less than 30 % from dietary fat and less than 10 % from saturated fat. For the PA component, participants were instructed to aim for at least 50 min/week of PA, then to increase this to at least 175 min/week by week 26) Usual care group (3 group meetings per year where diet, PA, and social support were discussed)	4 years	Changes in PA: Yes. Participants in the Intensive Lifestyle Intervention program increased their leisure time PA compared to the usual care group at year 4 Other outcomes: Participants in the intervention lost 4.9 % of initial weight, and increased their fitness compared to the usual care group at year 4
Jemmott III et al. [57]	RCT (with 6 and 12 months follow-ups post-program end)	1,181 Men from 22 residential neighborhoods 18–45 years South Africa	Social cognitive theory and theory of planned behaviour	Male-only sample and male-centered approach The program was gender and culturally sensitised based on formative research. Group sessions included male-oriented activities such as beginning with a circle of men and brainstorming activities about what it means to be a man	Group-based Face-to-face	Self-reported PA, fruit and vegetable consumption, and alcohol consumption	The clustered RCT included two groups: The health-promotion intervention (delivered by a male facilitator this intervention included six 75-min group sessions delivered over 3 consecutive weeks. Sessions involved interactive activities with games, brainstorming, and videos focused on improving PA, fruit and vegetable consumption, and reducing alcohol and fat intake, along with practicing PA to increase self-efficacy) The HIV/STI risk-reduction intervention group (participants in this group served as a control group, and also attended group sessions involving interactive activities delivered by a male facilitator with a focus on HIV/STI risk reduction)	3 weeks	Changes in PA: Yes. Participants in the health-promotion intervention group significantly increased their moderate-vigorous PA compared to the control group participants (averaged over 6 and 12 month follow-ups) Other outcomes: No changes in fruit and vegetables intake or alcohol consumption
Kim et al. [65]	Pre-post	138 People with metabolic syndrome (83 men) 40+ years Korea	No	Co-ed	Individual-based Print-based Internet-based Face-to-face	BMI, weight, WC, SBP, DBP, fasting plasma glucose, insulin, TC, TG, HDL-C, plasma adiponectin levels, and plasma vaspin levels	Individually tailored lifestyle modification program (focused on achieving a balanced diet. Additionally, participants were given an individualized exercise plan. Participants were also encouraged to quit or reduce smoking and alcohol use. Participants received bimonthly individual counselling as well as motivational e-mails three times a week with information on healthy eating and PA)	10 months	Changes in PA: Could not determine Other outcomes: Significant favourable changes in metabolic factors (SBP, DBP, TC, TG, adiponectin, and insulin) from pre- to post-intervention. BMI, weight, and WC were unchanged by the intervention
Lubans et al. [48]^a^	RCT pilot study (with 3 months follow-up post-program end)	53 Overweight or obese fathers and 71 school aged children Mean age: 40.6 years Australia	Social cognitive theory and family systems theory	Male-only sample and male-centered approach Drew on men’s motivation to be healthy role models for their children, and the program included gender sensitive messages and materials	Group-based Face-to-face	Father’s weight, father’s PA, and father’s dietary intake	Healthy Dads Healthy Kids (HDHK; fathers attended 8 weekly group sessions. Five of these sessions were only attended by fathers while the other three included fathers and their children. Each of the eight sessions was 75 min in length. Fathers were given evidence based information surrounding the benefits of weight loss and behavior change. Fathers were asked to model these behaviors for their children. Fathers were encouraged to interact with their children through the medium of healthy eating and active living)	12 weeks	Changes in PA: Yes. Fathers’ in the intervention group increased their PA (total number of steps per day) compared to fathers’ in the control group Other outcomes: Weight loss in the intervention group was statistically significant. Increases in PA mediated the effect of the intervention (i.e., fathers who increased their PA decreased their weight)
Maruyama et al. [59]	RCT	101 Male office workers who had metabolic syndrome risk factors 30–59 years Japan	No	Male-only sample	Individual-based Internet-based Face-to-face Workplace-based	Changes in food group intake, steps taken, BMI, umbilical circumference, BP, and blood analysis	Life Style Modification Program for Physical Activity and Nutrition program (LiSM10! monthly counselling sessions with dietician and physical trainer, enter weight, food intake, PA and pedometer data on website. Family members and health counsellors could view and comment on web entries) Control group (no treatment)	4 months	Changes in PA: No. No difference in number of steps Other outcomes: Significant treatment effects for parameters related to insulin resistance, BMI, umbilical circumference, and habitual food intake
Morgan et al. [50]	RCT (with 3 months follow-up post-program end)	159 Overweight and obese adult men 18–65 years Australia	Social cognitive theory	Male-only sample and male-centered approach The SHED-IT program was designed specifically for men	Individual-based Internet-based Paper-based	Body weight, BMI, WC, body composition (e.g., visceral fat), BP, RHR, PA, sedentary behaviors, dietary intake, food portion size, alcohol consumption, quality of life, and sleepiness	Self-help, Exercise, and Diet using Information Technology (SHED-IT) community trial included three groups: The SHED-IT resources group (provided with resources geared specifically toward men and based on social cognitive theory, including: a weight loss DVD, a weight loss handbook, a pedometer, a tape measure and a kilojoule counter book) The SHED-IT online group (received all of the same resources as the SHED-IT resource group, as well as a website directory, online food and exercise diaries, and 7 feedback e-mails about their online entries) Control group (no intervention until after the 6 months assessments)	3 months	Changes in PA: Yes. Men in both intervention groups had significantly greater improvements in PA compared to those in the control group Other outcomes: Men in both the resources and the online treatment groups lost significantly more weight than men in the control group (3.2 and 4.2 kg more than control group, respectively). The weight loss difference between the resource and online group was not significant. Men in both intervention groups had a significant decrease in BMI, SBP, WC, visceral fat, alcohol consumption, and significantly greater improvements in quality of life compared to those in the control group. The reduction in WC was greater for the online group than the resources group. Differences in all other secondary outcomes were not significantly different between intervention groups
Morgan et al. [49]	RCT (with 7 weeks follow-up post-program end)	93 (18–65 years) overweight or obese fathers (average BMI 32.5) and 132 school aged children Australia	Social cognitive theory and family systems theory	Male-only sample and male-centered approach The program drew on men’s motivation to be healthy role models for their children, and the program included gender sensitive messages and materials	Group-based Telephone-based Face-to-face	Fathers body weight, WC, BMI, RHR, BP, and sitting time	HDHK participants were randomly assigned to be a part of either the intervention group or the control group. Fathers in the intervention group attended 7 group based sessions that were held weekly for 90 min per session. Four of these sessions were administered only to fathers and the remaining three sessions involved both the fathers and their children. The sessions were intended to teach fathers about how healthy eating and PA could be a good way for fathers to spend time with their children. Sessions involving children focused on PA	7 weeks	Changes in PA: Yes. Increased fathers’ PA Other outcomes: Fathers in the intervention group lost significantly more weight and showed a significant decrease in WC and BMI compared to fathers in the control group. There was not a significant difference between the control group and the intervention group’s amount of sitting time or BP
Morgan et al. [51]	RCT	110 Overweight or obese men 18–65 years Australia	Social cognitive theory	Male-only sample and male-centered approach The Workplace POWER program was based on the gender-sensitive SHED-IT program, modified to be more appropriate for male shift workers	Group-based Internet-based Print-based Face-to-face Workplace-based	Weight, WC, BMI, BP, RHR, PA and dietary variables, PA and dietary cognitions, level of PA, and dietary intake	The Workplace POWER program: Preventing Obesity Without Eating *like* a Rabbit (involved one 75 min information session delivered by a male researcher followed by a 3 months online component. Participants entered weight, eating and exercise data and received individualized feedback via email. Participants were also provided a weight loss handbook, website user guide, pedometer, and offered a group-based financial incentive) Control group (wait-list)	12 weeks	Changes in PA: Yes. Significant treatment effect on PA reported Other outcomes: Significant treatment effects for weight, WC, BMI, SBP, RHR, PA and some PA cognitions No treatment effect for most dietary variables 28 % adherence to online component
Morgan et al. [66]^a^	RCT (with 3 and 9 months follow-ups post-program end)	65 Overweight or obese men that were either students or staff members at the University of Newcastle 18–60 years Australia	Social cognitive theory	Male-only sample and male-centered approach The SHED-IT program includes gender-sensitized materials	Individual-based Internet-based Face-to-face	BMI, WC, and BP	In the SHED-IT program participants were randomly assigned to one of two groups: Control group (information presented face-to-face and in a weight loss booklet: no website access granted) The intervention group (one face-to-face session, weight loss booklet, and website access. The intervention group monitored their weight, amount of PA, and dietary intake online. The intervention group were allowed to post questions online which were answered on a weekly basis by one of the researchers)	3 months	Changes in PA: Could not determine. In this intervention PA was not measured as an outcome Other outcomes: Both the intervention and control group lost weight from baseline to the 12 month follow up (amount not significant between groups). Improvement in BP and WC in both groups. SBP was improved significantly more in the intervention group than the control group. There was a significant, positive correlation between the amount of weight lost in the intervention group and the number of daily exercise entries made online. This relationship was present for weight, diet, and exercise entries
Patrick et al. [52]	RCT	441 Overweight and obese adult men 25–55 years United States	Social cognitive theory	Male-only sample and male-centered approach The program was tailored to men based on interviews with men’s weight loss experts and feedback on the program content from focus groups with men	Individual-based Internet-based	Weight, WC, BMI, diet, and PA	Weight loss intervention (participants received a computerized assessment to create recommendations for behavior change, weekly web-based learning activities, and weekly individualized feedback on their progress administered through the internet. The participants completed online assessments and set goals for change and their weekly progress was displayed graphically on the web site. Participants were also allowed to e-mail experts (e.g. a dietician) with any health related questions. The intervention group was given a pedometer to wear and encouraged to log their steps as well as PA minutes where they could not wear the pedometer) Control group (wait-list)	1 year	Changes in PA: Reported walking was increased in the intervention group Other outcomes: No significant difference in BMI, WC or weight between the intervention and control groups after the 1 year intervention. The intervention group showed a positive change in dietary behavior (an increase in fiber, fruit, and vegetable intake)
Pringle et al. [53]	Pre-post	1,159 Adult men who were enrolled through advertising, promotional events, and outreach programs 18–44 years England	No	Male-only sample and male-centered approach The program engaged men through football clubs and was delivered by health professionals with training specifically in men’s health promotion	Group-based Face-to-face	PA, diet, unemployment, and substance use	National men’s health program delivered by/in English Premier League (EPL) football clubs (the intervention included engaging in PA as a group (e.g. playing football) and health promotion. 16 football clubs participated in this study and there was no standardized intervention. Clubs based their intervention on a needs assessment for the community they were located in. Activities led by health trainers in each football club)	3 months	Changes in PA: Yes. The men showed a significant increase in their level of PA. Other outcomes: There were also significant increases in fruits and vegetable intake. Further, men showed a significant decrease in their BMI, alcohol consumption, and sedentary time
Sealey et al. [67]	Pre-post Pilot study	24 Male supporters of a rugby league or rugby union club 35–65 years Australia	No	Male-only sample and male-centered approach The program was influenced by the FFIT program and was delivered at a rugby sporting club	Group-based Face-to-face	Physical and mental health, BMI, and WC	Pilot intervention (Week 1: introduction and baseline data collection, Week 2–11: 45 min exercise session and 45 min educational session once/week, Week 12: motivational session. Exercise sessions were supervised by Sport and Exercise Science University students and included a warm up, brisk walk, circuit exercises, and cool down. Educational sessions were interactive and included content on men’s health issues. Participants were also provided with a pedometer, tape measure, and step diary and were encouraged to exercise individually for at least 30 min/day)	12 weeks	Changes in PA: Could not determine Other outcomes: No significant improvement in healthy lifestyle knowledge Significant reduction in WC and improvement in physical and mental health in both the rugby league and rugby union club groups. Significant changes in BMI were seen only in the rugby league group Results from focus group sessions post-intervention indicated various health and lifestyle improvements in participants Change in body composition for half of participants resulted in a decreased risk of disease
Werkman et al. [60]	RCT (with 1 year follow-up post-program end)	415 recent retirees (352 male) 55–65 years The Netherlands	No	Co-ed	Individual-based Print-based Internet- based Face-to-face	Body weight, BMI, WC, arm, hip, thigh, and calf circumference, abdominal sagittal diameter, body fat, SBP, DBP, PA, and dietary intake	Energy balance program (aimed at small and sustained PA/diet changes, 5 program modules, including toolbox with information, pedometer and waist tape, CD-ROMs with tailored feedback on BMI, PA, and diet, study website and online forums, interactive weight maintenance program, and general newsletters) Control group (general newsletters and general study information)	1 year	Changes in PA: No. PA improved from baseline, but intervention group was not significantly different than control Other outcomes: Decreased WC, weight, BMI, SBP, DBP and improved PA and dietary behavior from baseline to follow-up. The beneficial effects were seen to a greater extent in intervention group, compared to controls, but between group differences were not statistically significant However, among participants with a lower level of education, WC and fat intake were significantly decreased at 12 month follow-up compared to control group
Zwolinsky et al. [54]	Pre-post	130 men that were recruited to participate through unemployment agencies, community centers that provided services to people who are considered to have a low socioeconomic status, and drug rehabilitation centers 18+ years England	No	Male-only sample and male-centered approach The program was delivered through English Premier League football/soccer clubs	Group-based Face-to-face	Diet, PA, alcohol intake, and smoking cessation	The intervention consisted of weekly free classes (composed of 1 h of exercise, health checks and behavioral counselling meant to improve the participants self-monitoring. This was supplemented with health seminars that were intended to increase knowledge about the risks associated with unhealthy behaviors)	12 weeks	Change in PA: Yes Other outcomes: 19 % of men changed one health behavior, 35 % of men changed two health behaviors, and 67 % of men changed 2 or more health behaviors. Diet and PA were the two primary lifestyle behaviors that were improved upon by men. It was shown that men who were employed were five times more likely to increase their level of PA at the 12-week follow up. Further, if the participant improved their diet they were twice as likely to increase their PA to over 150 min of exercise a week

*BMI* body mass index, *BP* blood pressure, *Co-ed* coeducational, meaning open to both sexes, *DBP* diastolic blood pressure, *GP* General Practitioner, *HbA1c* hemoglobin A1c, *HDL-C* high-density lipoprotein cholesterol, *HR* Heart rate, *LDL-C* low-density lipoprotein cholesterol, *PA* physical activity, *Pre-post* pre-intervention assessments and post-intervention assessments were conducted, *RCT* Randomized controlled trial;, *RHR* resting heart rate, *TC* total cholesterol, *TG* triglycerides, *SBP* systolic blood pressure, *VO*_*2peak*_ peak volume of oxygen, *WC* waist circumference

^a^Study was published after the George et al. [30] review but is based on the same sample as a study covered by the George et al. [30] review

## Findings

The 35 studies included in this review involved 31 different programs and a total of 14,383 male participants (sample sizes from 24 to 4,870). It is also important to note that seven of the 35 studies reported the results of pilot programs.

The majority of the studies (*n* = 24, 69 %) evaluated programs that were only offered to men. Male-only programs were offered in 7 of the 13 studies that evaluated physical activity only programs (duration 8 weeks to 2 years). Of the 22 studies that included programs designed to enhance physical activity in combination with other health behaviors (duration 7 weeks to 5 years), 17 had male-only samples. The primary outcome of interest in the present review was change in physical activity, but secondary outcomes indicative of changes in physical activity were also of interest. Among the studies meeting the inclusion criteria (*n* = 35), 24 reported significant increases in men’s physical activity [34–57] and three reported no changes in physical activity [58–60]. For the remaining eight studies, changes in physical activity could not be determined because this was not directly assessed as an outcome variable [61–68]. However, in these studies changes in secondary measures (e.g., cardiovascular health, metabolic factors) suggested positive changes in physical activity. Of the 35 studies reviewed, 12 involved follow-up assessments at time points after intervention completion (ranging from 7 weeks to 15 months post-program end) [34, 40, 45, 46, 48–50, 57, 58, 60, 61, 66]. All but two [58, 60] of these demonstrated successful maintenance of physical activity and/or secondary measures indicative of positive changes in physical activity; however, follow-up durations were primarily shorter: in nine studies the follow-up was conducted less than 1 year following the conclusion of the program [34, 40, 45, 46, 48–50, 58, 66], in two studies follow-up assessments were conducted 1 year after the program conclusion [57, 60], and in one study the follow-up was conducted at 15 months after the program conclusion [61].

Because some of the included articles were separate studies based on the same program (e.g., Healthy Dads, Healthy Kids), the findings are reported based on the 31 distinct programs, rather than the 35 articles. In total, 28 of the 31 separate programs were successful in increasing either physical activity or a secondary measure indicative of increases in physical activity. The findings are summarized below organized according to theoretical approach, mode of delivery, strategies for physical activity promotion, and gender-sensitive programs.

### Theoretical Approach

Of the 31 different programs, 14 explicitly described theoretical perspectives guiding behavior change approaches [34, 38, 40, 41, 48–50, 52, 55, 57, 58, 68–70], the most common being social cognitive theory [71], which guided 12 different programs, often in combination with other theoretical approaches. Overall, theoretical constructs were measured in three of the studies reviewed. Andersen et al. [34] found that social support, self-efficacy and outcome expectancies among men increased in the program group compared to the control group; however, the changes in these social cognitive constructs were unrelated to changes in physical activity. Although Gram et al.’s [69] program was not explicitly based on the theory of planned behavior [72], post-intervention interview data suggested program adherence (exercise behavior) fit within a theory of planned behavior model (i.e., positive attitudes about exercise, a sense of obligation or social pressure, and perceptions of control about ability to succeed influenced exercise behavior). Finally, although the theory of planned behavior did not guide Sheeran et al.’s [40] program, they found that constructs from the model helped explain how mental contrasting enabled participants to more effectively translate their attitudes about the importance of physical activity into action (i.e., engaging in physical activity) compared to a control group.

### Mode of Delivery

The programs included in this review varied in terms of modes of delivery. Although some programs were offered to both men and women (*n* = 11), the majority were men-only programs (*n* = 20). Programs offered opportunities for structured/supervised physical activity in group sessions (usually combined with recommendations that participants complete additional physical activity individually) [34, 35, 38, 43, 45, 46, 48, 49, 53, 54, 57, 61, 64, 67, 70], in individual, face-to-face sessions (e.g., with a personal trainer) [62, 63], or simply encouraged participants to exercise on their own [39–42, 44, 47, 50–52, 55, 56, 58–60, 65, 66, 68, 69].

In some programs a variety of resources were also used to support program delivery (e.g., print-materials, DVD, tracking tools, regular personal reports on adherence; email prompts/motivational messages). Web-based platforms were among the more innovative approaches used for elements of program delivery and also formed a medium for self-monitoring and peer support. In total, eight different programs were evaluated that involved internet components [44, 50–52, 55, 59, 60, 66, 70]. Within these programs, variability existed in how the internet was utilized. For example, in a weight loss program, the Self-help, Exercise, and Diet using Information Technology (SHED-IT) program [50, 66], the online enhanced delivery approach included a website directory, online food and exercise diaries, and feedback emails and was compared with a group that received a print-based resource. In five other programs the internet was used to facilitate monitoring of physical activity and pedometer data, food intake, and/or weight [44, 52, 55, 59, 60]. In one of these internet programs, family members and counsellors were also able to view and comment on progress [59]. In another, an interactive website and mobile phone web app provided a medium for connecting with male peers as a means of promoting social support and friendly competition [55]. The internet was also used as a medium for friendly competition in a corporate team-based program where teams of men and women were able to compare their progress and communicate with team members using a virtual platform [44]. Taking full advantage of the opportunities to develop computer-tailored individualized programs, one group designed a low intensity energy balance online program for recent male retirees (average age 59.5 years) to provide tailored advice. However, full exposure to the program was low in that less than 50 % of participants in the program utilized the online modules and resources [60]. In contrast, engagement in a 12 month, web-based program for overweight and obese men was more encouraging [52]. This program was specifically tailored to men’s preferences and was flexible. For example, men made their own choices regarding behaviors to work on at any particular time. On average the men in the program (average age 43.9 years) logged onto the website to set weekly goals for 23.4 weeks (SD  =  16.7) over the duration of the program.

The setting was also identified as an important consideration in engaging men in physical activity programs. In this review there was a marked increase in the types of entry points and strategies for engaging men in physical activity compared to the studies reported by George et al. [30]. A few researchers leveraged the workplace as an intervention location [39, 44, 51]; however, four other programs to support men’s physical activity traded on men’s interest in sports by engaging men through their affiliations with organised sports and delivering group exercise sessions at local sports clubs [45, 46, 53, 54, 61, 67]. One additional program did not actually engage men in group exercise, but did recruit participants through a local angling club [40]. Although incentives were offered in a few programs—such as free access to a gym [42] and group-based financial incentives [51]—friendly competitions or challenges were also used to engage and motivate male participants [38, 44].

In the few studies that were not successful in demonstrating significant increases in physical activity, the programs varied considerably [58–60]. One similarity among these was that all used an individual approach, recommending participants engage in physical activity on their own. Maruyama et al. [59] noted that environmental barriers (e.g., lack of access to exercise facilities) may have led to their null results and suggested that such barriers may need to be addressed in future programs. Indeed, other programs that used an individual approach and provided free access to facilities were successful in increasing men’s physical activity [42].

### Physical Activity Promotion: Techniques and Approaches

The programs included in this review were based on various physical activity recommendations. For example, some programs encouraged participants to engage in 30 min of physical activity per day [41, 57, 67, 68], whereas in others participants were asked to increase their step count to 10,000 per day [42, 44, 52, 56] or aim for 10 miles of fast walking or jogging per week [64]. All programs focused on increasing cardiovascular or aerobic physical activity, and a few also included strength-building exercises [34, 45–47, 55, 57, 70]. In one program, participants’ adherence to the strength-building guidelines of two or more days per week was evaluated, and although intervention participants engaged in more moderate and vigorous activity compared to the control group, they did not differ in terms of strength-building activities [57]. Overall, the suggested amount of recommended moderate-vigorous physical activity ranged from 50 to 250 min per week.

In total, 13 programs included regularly scheduled physical activity. For example, men were engaged in group-based exercise sessions in 11 programs [34, 35, 43, 45, 46, 48, 49, 53, 54, 57, 61, 64, 67, 70], and in two other programs they followed individualized exercise plans supervised by personal trainers [62, 63]. In the remaining programs [38–42, 44, 47, 50–52, 55, 56, 58–60, 65, 66, 68, 69], participants were encouraged to increase their daily physical activity independently (without structured sessions). Whether physical activity was regularly scheduled or not, in most programs participants were encouraged to walk or run. In five of the programs reviewed, team-based sports such as football (soccer) were included [34, 45, 46, 53, 54, 61, 67].

Devices were used to assist men in monitoring their activity in 18 programs; including pedometers (*n* = 14), accelerometers (*n* = 2), and/or heart rate monitors (*n* = 2) [34, 42–45, 48–52, 55, 56, 58–61, 67–69]. Although results were generally favourable, 3 of the 13 programs that included pedometers for self-monitoring failed to demonstrate an increase in men’s physical activity [58–60]. In these programs no supervised exercise sessions were included. In contrast, four programs that used accelerometers/pedometers plus supervised group physical activity resulted in an increase in physical activity [34, 43, 45, 48, 49].

### Gender-Sensitive Physical Activity Programs

Though 20 of the programs were offered exclusively to men, it did not appear that sex or gender-related factors informed the design or delivery in eight of these programs [34, 39, 40, 42, 43, 56, 59, 69]. In the majority of cases, the programs were tailored to theory-based considerations (e.g., self-efficacy, social support) or personal risk estimates, and based on accepted recommendations regarding physical activity for healthy adults. If face-to-face counselling was used, gender-neutral strategies, such as goal-setting and providing advice, predominated [34, 39, 42, 43, 59]. However, the remaining 12 programs offered exclusively to men demonstrated an emergence of innovative approaches to promoting physical activity that acknowledge men’s interests and preferences (i.e., that are gender-sensitive) in a variety of ways. What is common among these programs [38, 45, 46, 48–55, 57, 61, 66, 67, 70] is that they are designed from the ground up as sex-specific and gender-sensitive programs.

Four similar programs to encourage participation in physical activity by drawing on men’s interest and involvement in football (commonly referred to as soccer in North America and Australia) were evaluated and found to be highly successful in increasing physical activity as well as other health behaviors [46, 53, 54, 61, 67]. These programs were based in England, Scotland and Australia where football/soccer has a long-standing and dedicated male fan base. Delivered through football/soccer clubs, the programs engaged coaching staff at the clubs or qualified trainers and involved men in a variety of group exercises. Football Fans in Training (FFIT) [45, 46], an example of this approach, offered a 12 week “gender-sensitized” program focused on weight loss, physical activity, and healthy eating advice to overweight and obese men and demonstrated improvements in self-reported physical activity and healthy eating. Underlying the success of this approach is men’s familiarity and comfort with the setting (i.e., football clubs) and an approach that works with masculine ideals rather than against them. Designed to be appealing to male football fans, the FFIT program included club-based incentives (e.g., club T-shirts, visits from club celebrities), friendly competition, education related to alcohol consumption, and the use of “banter” in discussions of sensitive health issues (e.g., weight gain) [45]. Comparable programs delivered in the UK and Australia also produced encouraging results [53, 54, 61, 67].

An Australian based group has developed three programs—each with unique aspects and program names designed to appeal to men. The SHED-IT program was specifically designed to promote healthy lifestyles and facilitate weight loss among men [50, 66]. In recognizing that men were not attracted to structured, face-to-face programs, alternatives were developed that would not involve face-to-face contact. The SHED-IT resource package consisted of DVDs and handbooks along with a pedometer, tape measure for waist circumference, and a kilojoule counter book. Access to an online food and exercise diary site was also provided to one group of SHED-IT participants to assess the efficacy of the paper-based resources compared to augmenting resources with the online support [50]. Although the SHED-IT program was based on Bandura’s [71] social cognitive theory (e.g., self-efficacy, self-management, perceived barriers, and social support), the resources were tailored specifically for men, utilising dynamic culturally sensitive language affirming masculine virtues in order to engage Australian men (e.g., “Weight Loss Handbook for Blokes”). A light-hearted and sometimes humorous approach to physical activity and dietary behaviors was used to reflect men’s applied and active approaches amid ensuring autonomy in establishing their own goals for the program. In these and other ways, the messages, their delivery, and affirmation for participating avowed an array of masculine virtues. Although the pilot study results for SHED-IT [73] were reported in the previous systematic review by George et al. [30], subsequent RCT results indicate that the online and the resource-based programs were equally effective in improving physical activity and promoting weight loss compared to the control group [50].

The second program, Preventing Obesity Without Eating Like a Rabbit (POWER), was a workplace-based weight loss program designed to engage overweight and obese male shift workers [51]. Modelled after SHED-IT, the Workplace POWER program was tailored for shift workers and included information sessions or DVD resource, an interactive study website, and group-based financial incentives to improve healthy lifestyles with respect to physical activity and healthy eating. Workplace POWER was a men-only program, and the setting, mode, and delivery mechanisms were designed to appeal to men. The use of prescriptive rules about diet was avoided. Instead, core messages were about how to fit in more physical activity and healthy eating with minimal disruption to daily life and assurances that they didn’t have to give up things (e.g., beer and wine) to lose weight. Results indicate that the Workplace POWER program was effective in decreasing body weight and increasing physical activity.

The third program developed and evaluated by this group of Australian researchers was the Healthy Dads Healthy Kids (HDHK) program [48, 49]. This program was a 7-week healthy lifestyle program designed for overweight/obese fathers and pre-school children delivered in community settings by trained male facilitators. Capitalizing on ideals related to fathering, healthy eating and physical activity were positioned as good ways for fathers to spend time with their children. In health-related information and physical activity sessions that traded on men’s interest in being good fathers, men were provided with the knowledge, skills and encouragement to model health behaviors in their role as fathers. In addition to physical activity sessions for fathers, several group sessions involved both children and fathers, focusing on aspects of father/child activity such as rough and tumble play and fun and interactive games. The face-to-face program was supplemented with resources including a physical activity handbook, a weight loss handbook for men, and a website to support self-monitoring of weight, exercise and dietary intake during the program. The program demonstrated improvements in physical activity and other health behaviors among both fathers and children.

Another Australia-based program, ManUp, was a 9-month information technology-based intervention to promote physical activity and healthy eating among middle-age males [55]. In this program “ManUp Challenges” to increase levels of physical activity and healthy eating were offered using web and mobile phone based technology. With these challenges, men were provided with educational materials, tools for goal setting and self-monitoring, and automated feedback on progress. To cater to varying levels of physical activity and dietary habits, build confidence, and provide opportunities for progressive changes, men could choose “light,” “mid” or “full” strength ManUp challenges. In addition, the intervention was designed to encourage online social support by providing participants with the opportunity to view and comment on the progress of others, and challenge their friends. Similar to the SHED-IT program, the information-technology and the print-based intervention arms of the ManUp program were equally effective in improving physical activity among men.

Another program was a 3-week health promotion intervention specifically tailored for South African men (18–45 years of age) [57]. In small groups of 9–15, the short community-based program was delivered by a male facilitator in the native language (isiXhosa) and involved male-centered activities. For example, the sessions began with a “circle of men” to foster a bond as “brothers” where participants could express their feelings openly and without judgement. The sessions also involved interactive activities with games, role plays, and videos focused on improving physical activity, fruit and vegetable consumption, and reducing alcohol and fat intake. Men also practiced strength-building and aerobic exercises to increase self-efficacy and were encouraged to engage in a combination of these exercises on a weekly basis. Lastly, the men participated in a brainstorming activity which focused on what it means to be a man and how men can influence their own health as well as the health of their families and communities. Although brief, this program was effective for increasing men’s physical activity at 6 and 12 month follow-ups compared to a carefully matched control group.

The remaining three examples of gender-sensitive programs were based in the US. The first, a 1-year internet-based weight loss program for men [52], was based on social cognitive theory, feedback from focus groups conducted with men, and interviews with two experts in men’s weight loss. The program was tailored to men’s preferences for a focus on “the facts” (and not feelings) and a “to-do” list with links to more detailed information underpinning the program. In addition, short weekly web-based learning modules were designed using “business like” language and graphics and men were encouraged to set realistic weekly goals and report progress toward their “to do” list online. Autonomous decision making was also supported by allowing men to choose the behaviors to work on. Improvements in physical activity and diet were observed among those with the highest levels of adherence to the program. Finally, two innovative gender-sensitive pilot programs designed to engage African American men in physical activity were evaluated [38, 70]. Hooker et al. [38] drew on previous research with mid-life African American men to integrate cultural and gendered components into a tailored 8-week group program to promote physical activity behavior change. Traditional views of masculinity and role perspectives were integrated into the program and reflected in concepts such as responsibility, stress management, and relapse prevention. In addition, activities to promote camaraderie were included as well as friendly team competition and a community service project. Men were assisted by the two trained facilitators to set overall and weekly physical activity goals, the majority of which were completed outside of the group sessions.

Also drawing on previous research with African American men [37, 74, 75] and informed by Hooker et al.’s [38] work, Griffith and colleagues [70] developed a community-based program called Men on the Move. Underpinned by social cognitive theory and self-determination theory, the aim of the program was to improve access to age and ability-appropriate, male-focused physical activity opportunities for African American men. Small group weekly workouts to foster male peer support and provide opportunity for modeling of exercise by other men of similar age were offered under the direction of a male fitness trainer. These sessions were augmented with information about community resources (e.g., schedule of fitness classes), handouts to guide home stretching and exercising, a list of helpful websites related to physical activity, and encouragement to contact others in the group to exercise together between sessions. The evaluation results of both of these pilot programs provide support for the feasibility of these new initiatives and potential for promoting men’s health when programs address their needs and interests.

## Discussion

The findings of this review extend knowledge related to promoting men’s physical activity and provide directions for future research. Since the review conducted by George et al. [30], there has been growing interest in designing and evaluating programs to promote men’s physical activity with 35 new studies published within the last 5 years. The majority of studies in this review reported significant increases in physical activity. It is possible these programs may also produce mid- or long-term changes in physical activity; however, the availability of only a few studies with long-term follow-up limits clear conclusions on sustained effectiveness [34, 35]. The included studies were characterized by a wide variety of programs, many of which hold potential for increasing men’s physical activity. This is consistent with previous work insofar as it points to the general effectiveness of programs that have some component of individual tailoring or that involve some degree of personal contact. However, given the multi-component nature of these programs, it remains unclear what unique variability each component contributes to positive behavior change. The features of program delivery were, to a large extent, constrained by the features that authors typically report and that can be easily and objectively verified (e.g., whether email messages were used). There may be other important features that are not routinely collected or reported, or that are difficult to measure. As internet-based programs and smart-phone applications become more common, it should be relatively easy to integrate these additional delivery features into current programs—particularly for young and middle adult men who are familiar with these technologies. Nevertheless, despite the unique challenges of engaging men in health promotion and the gender-related factors influencing men’s engagement in health behaviors [15–17], only 12 of the programs considered these factors in the design and delivery of approaches to promote men’s physical activity.

The results of the current review indicated that overall, even with calls for theoretically-driven health promotion programs to enhance effectiveness [76], explicit linkages were only examined directly in three of the studies making it difficult to draw conclusions about the role of theory-based strategies for increasing physical activity among men. Furthermore, many different strategies supporting behavior change have theory-based underpinnings making it possible that programs without explicitly stated theoretical underpinnings also had an underlying theoretical basis. In the wider literature on physical activity interventions the potential role of either theoretical constructs or specific behavior change strategies is also rarely examined [77]. Others have called for more research to examine the potential mediating role of theoretical factors in the success of interventions [76, 77], and it seems timely to also suggest that researchers ought to investigate the potential role of gender-related factors within the effectiveness of theory-based strategies for behavior change.

There may also be a need for new theoretical foundations for men’s health promotion programs. Although the connections between sports and men’s hypercompetitive and homosocial practices have been critiqued, men’s willingness to do physical activity appears to be a central consideration to successful health promotion efforts [78]. Indeed, of all the facets of health promotion, physical activity prevails as most likely to engage men with their health [79]. In this regard, engaging men in physical activity draws upon as well as provides opportunities to garner masculine capital by affirming competitiveness and/or striving for physical prowess [80]. Oliffe et al. [81] name this as a strength-based approach to men’s health promotion whereby rather than seeking to change men, some masculine norms (i.e., willingness to engage physical activity) are deliberately targeted to catalyze men toward self-health. Within the literature reviewed here it is fair to say that physical activity masculine norms are, at best, implicit. While this might be an oversight or triaged as relatively unimportant next to the empirical findings, there are good reasons to cultivate the principles underpinning men’s involvement with physical activity based health promotion. For example, men’s health promotion program design may be reliably informed by what has worked for others as well as why that might have been the case. Conversely, much can be learned about what to avoid by distilling what fails to invigorate men in the context of physical activity centric health promotion programs (e.g., mixed gender programs, group versus personal trainer models). In this regard, the barriers to men’s engagement along with principles underpinning participation should be understood as carrying equal empirical weight. After all, these collective insights are key to advancing the application of health promotion theories to men-centred program design.

In the present review, the mode of delivery of physical activity was integral to the success of a program at increasing physical activity. It is noteworthy that all studies that involved men engaging in physical activity with other men through professional sports resulted in increased physical activity. Although group exercise was limited to 1–2 h per week, men may have gained the motivation to exercise individually from these sessions. These findings support the suggestion by George et al. [30] that participating in sports teams can “increase adherence and enhance motivation” in men. Furthermore, contrary to evidence suggesting that pedometers can increase motivation [82], several (although not all) of the programs that provided participants with pedometers were unsuccessful in increasing physical activity among men, mirroring George et al.’s [30] findings. It may be that a focus on strength-building in combination with vigorous activity to increase overall fitness may be more appealing to men in the sense that it is more aligned with masculine ideals related to health and exercise.

Several of the programs included in this review were designed for obese and overweight men and as such not only aimed to increase physical activity but also to enhance healthy eating. Although physical activity was often reported as the drawing card, once in these programs men were responsive to and interested in nutritional information when it was tailored to them. The lessons learnt from these programs are instructive. For example, in the Workplace POWER trial, researchers found that blue collar working men preferred very specific food-based guidelines such as “Eat this or don’t eat this” or “Drink less” [51, 83] and responded favourably to humor that protected masculine connections with food (e.g., “you don’t have to eat like a rabbit”) [51, 84]. Thus, male-specific advice and information appears to be an effective strategy for engaging men in weight loss and provides additional support for taking into account gender-related factors in designing programs to support men’s healthy living. These strategies suggest the potential for engaging men in a wide variety of health promoting behaviors beyond physical activity.

Also evident is that most men’s health promotion success stories—both in terms of feasibility and uptake—have been community-based rather than professionally produced programs [81, 85]. This trend might relate to men’s resistance and reluctance around engagement with professional health care services and/or providers. Yet, rather than continuing to debate that well traversed terrain about why men don’t go to the doctor, dividends reside in linking to end-users early on, both as a means to designing men-centred programs but also in regards to sustaining those programs (i.e., train-the-trainer models). After all, many a seemingly popular program has ended due to funding restraints, and the feasibility of health promotion programs increasingly resides with community-based champions—many of whom are unpaid but highly motivated.

Robertson et al. [33] outlined five themes evident in successful and sustainable health promotion work with men: (a) settings that facilitate men’s engagement (e.g., workplace or sports clubs); (b) using a gender-sensitive style or approach; (c) listening to and incorporating feedback from men, (d) providing adequate training and ongoing support; and (e) partnering with trusted community groups. In the present review, the programs that were considered to have taken a gender-sensitive approach drew on many of these themes in the design and delivery of their interventions. Overall, 16 of the studies (comprising 12 different programs) in this review explicitly integrated gender to the program design in some way, whereas only three studies in the original George et al. [30] review had a men-centered approach (and none of these were published prior to 2009). It is noteworthy that all 12 of the programs deemed to be gender-sensitive demonstrated either a significant increase in participants’ physical activity or other substantive improvements (e.g., weight loss). These programs provide a promising avenue for engaging men and hold the potential for sustainable health promotion in this hard-to-reach population.

Taken together, the findings reveal some optimising practices in the development and delivery of programs to promote changes in physical activity among men. Interventions may be delivered using group, individual or mixed modes (individual and group); there are examples of successful physical activity programs using each of these delivery modes. What is evident, however, is that programs may benefit from a diverse set of delivery platforms. Entry points for engaging men in physical activity were somewhat more diverse in the reviewed studies compared to those reviewed by George et al. [30] which focused mainly on community-based and health care provider referrals. In the present review, in a handful of studies novel strategies were employed, such as engaging male members of football and angling clubs, and engaging fathers to exercise with their kids. Yet aside from these novel strategies, the majority of physical activity programs for men are not accounting for emerging research on ways that men access and engage in health promotion activities. Although male-dominated workplaces can provide important sites for men’s physical activity programs, employers have traditionally focussed on workers’ compensation rather than targeted health promotion programs [86]. Increasing mental illness related absenteeism and presentism compensation might prompt employers to rethink the benefits of providing work-based programs, especially given the connections between physical activity and men’s mental health promotion. Even within the trend of employing health promotion consultants to deliver face-to-face and online resources to underserved worker groups, there is great potential for both advancing the work fitness of employees and reducing worker compensation payouts.

Clearly this is a field where continued research efforts are important to guide the development of effective physical activity programs for diverse male groups. Since the effectiveness of interventions may vary among sub-groups of men and be dependent upon the intersection of gender with age, ethnicity, socioeconomic status, and related factors, researchers need to fully document the characteristic features of interventions, implementation processes (including level of engagement and how sustained engagement was achieved), and the demographic profile of participants. Further research on how to engage diverse groups of men in health promotion programs that include physical activity is also needed. In addition to the studies included in this review, there are evaluations currently underway of physical activity programs designed for specific subgroups of men such as the unemployed [87, 88]. Other groups that would benefit from tailored physical activity programs are men whose work involves sedentary activity (e.g., truck drivers), those with chronic diseases including cancer, and indigenous men. International collaborations to evaluate innovative approaches in multiple sites and in a variety of contexts would also advance the field.

## Conclusion

There is an increasing need for and interest in health promotion strategies that effectively target men. Physical activity appears to provide an effective way for men to access health promotion and thus warrants further investigation. Innovations in physical activity programs that focus on masculine ideals and gender influences to tailor programs for men may provide useful strategies in promoting other areas of men’s health. This new focus on gender offers a platform for continued innovation in men’s health promotion.
